# Fatal SARS-CoV-2 Reactivation After Allogeneic Hematopoietic Stem Cell Transplantation for Severe Aplastic Anemia

**DOI:** 10.7759/cureus.87084

**Published:** 2025-07-01

**Authors:** Yuri Furuyama, Tatsuya Suwabe, Ayako Kawakami, Hodaka Yonezawa, Takayuki Katagiri, Kyoko Fuse, Yasuhiko Shibasaki, Takashi Ushiki, Jun Takizawa, Hirohito Sone, Masayoshi Masuko

**Affiliations:** 1 Department of Hematology, Endocrinology, and Metabolism, Faculty of Medicine, Niigata University, Niigata, JPN; 2 Department of Hematopoietic Cell Therapy, Niigata University Medical and Dental Hospital, Niigata, JPN; 3 Laboratory of Hematology and Oncology, Graduate School of Health Sciences, Niigata University, Niigata, JPN

**Keywords:** aplastic anemia, covid-19, hematopoietic stem cell transplant, sars-cov-2, virus reactivation

## Abstract

SARS-CoV-2 has been reported to potentially remain in the lower respiratory tract for some time after it is no longer detectable in the upper respiratory tract, and this could be a source of reactivation. Reactivation of latent viral infections, such as cytomegalovirus and Epstein-Barr virus, after allogeneic hematopoietic stem cell transplantation (allo-HSCT) is often a clinical problem. COVID-19 is caused by SARS-CoV-2 infection and has a high mortality rate in allo-HSCT recipients. However, little is known about SARS-CoV-2 reactivation following allo-HSCT. In this report, a patient with severe aplastic anemia first developed mild COVID-19 (day 0) with negative antigen test results on day 27. Three months later (day 97), the patient underwent allo-HSCT. Two months post-transplantation (day 157, i.e., five months after the initial infection), the patient developed rapidly progressive respiratory failure and was diagnosed with severe COVID-19. Since the patient was hospitalized and there was no obvious route of infection, we have concluded that reactivation of SARS-CoV-2, which had infected the patient five months earlier, occurred under an immunosuppressive state after allo-HSCT. Regarding allo-HSCT in patients who have previously developed COVID-19, careful monitoring using real-time reverse transcriptase-polymerase chain reaction (RT-PCR) to detect SARS-CoV-2 could be useful for detecting SARS-CoV-2 reactivation and providing early treatment to prevent fatal COVID-19.

## Introduction

Viral reactivation after allogeneic hematopoietic stem cell transplantation (allo-HSCT) is often severe and poses clinical challenges. COVID-19 is caused by SARS-CoV-2 infection and has a high mortality rate in allo-HSCT recipients [1]. SARS-CoV-2 has been reported to remain in the lower respiratory tract for some time after becoming undetectable in the upper respiratory tract and could be a source of reactivation [2]. Although there have been many reports of COVID-19 after allo-HSCT [3-4], there have been no reports of reactivation after allo-HSCT. We encountered a patient with severe aplastic anemia who developed fatal respiratory failure due to SARS-CoV-2 reactivation following allogeneic hematopoietic stem cell transplantation, five months after a prior COVID-19 infection.

## Case presentation

A 42-year-old man was diagnosed with stage 4 severe aplastic anemia. He was treated with rabbit anti-human thymocyte immunoglobulin (rATG) and eltrombopag. He had undergone thymectomy for myasthenia gravis at the age of 24 years and had been maintained on long-term corticosteroids and cyclosporine thereafter, even after the diagnosis of aplastic anemia. Four months after the rATG administration, the patient developed mild COVID-19 (designated as day 0). Given a low cycle threshold (Ct) value of 14.6 SARS-CoV-2 RT-PCR, indicating a high viral load, remdesivir treatment was initiated. He had previously received three doses of the SARS-CoV-2 vaccine, with the most recent dose administered approximately nine months before this infection occurred; however, further details were unavailable. On day 27, the patient’s SARS-CoV-2 antigen test results were negative. On day 64, the patient’s pancytopenia worsened, and he was considered a non-responder to rATG. At that time, his laboratory findings were as follows: white blood cell count 0.31 × 10⁹/L, neutrophil count 0.13 × 10⁹/L, lymphocyte count 0.09 × 10⁹/L, hemoglobin 7.7 g/dL, and platelet count 10 × 10⁹/L (Table 1). Chest computed tomography showed no abnormalities. Given the life-threatening nature of the patient’s condition, allo-HSCT was deemed necessary. Accordingly, he was admitted to the hospital on the day of pretransplant evaluation and management. On admission, the patient was afebrile, exhibited no symptoms of long-COVID, and tested negative for SARS-CoV-2 antigen. At the time, our institutional policy did not include routine SARS-CoV-2 RT-PCR testing for asymptomatic and antigen-negative patients; therefore, RT-PCR testing was not performed in this case.

**Table 1:** Summary of the hematological and serological examinations before allo-HSCT. The patient had severe aplastic anemia before allogeneic hematopoietic stem cell transplantation (allo-HSCT), accompanied by profound neutropenia and marked lymphopenia.  Notably, the patient had severe hypogammaglobulinemia due to long-term corticosteroid therapy for myasthenia gravis. In addition, profound lymphopenia was observed, which was attributed to prolonged steroid use and prior administration of anti-thymocyte globulin.

Parameter	Result	Normal range
White blood cell count	0.31 × 10^9^/L	3.30-8.60 × 10^9^/L
Neutrophil count	0.13 × 10^9^/L	1.70-6.37 × 10^9^/L
Lymphocyte count	0.09 × 10^9^/L	0.99-3.16 × 10^9^/L
Hemoglobin	7.7 g/dL	13.7-16.8 g/dL (male)
Platelet count	10 × 10^9^/L	158-348 × 10^9^/L
Reticulocyte	24 × 10^9^/L	Not reported
Immunoglobulin G	588 mg/dL	861-1747 mg/dL
Immunoglobulin A	97 mg/dL	93-393 mg/dL
Immunoglobulin M	30 mg/dL	33-183 mg/dL

**Figure 1:**
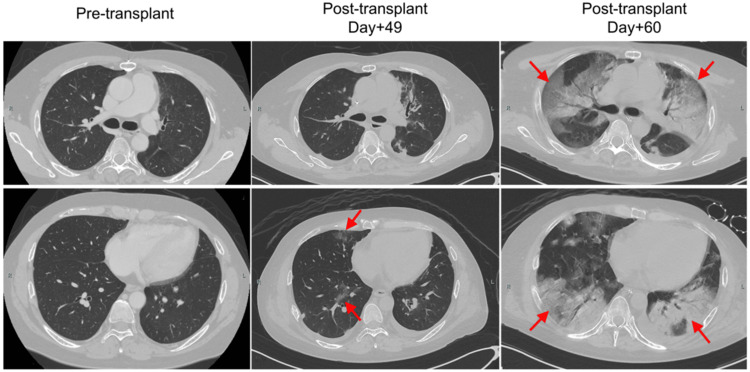
Serial chest CT images from pre-transplantation to the onset of COVID-19. The pre-transplant chest CT, performed on day 64 after the initial COVID-19 diagnosis, showed no abnormalities. On day 49 post-transplantation (day 146 after the initial infection), a CT scan performed due to mild hypoxemia revealed faint ground-glass opacities in both lower lobes. By day 60 post-transplantation (day 157 after the initial infection), CT revealed extensive bilateral consolidations with air bronchograms and a crazy-paving pattern in response to rapidly progressive respiratory failure. SARS-CoV-2 was detected by RT-PCR with a low Ct value, indicating active infection. Differential diagnoses, such as bacterial pneumonia, cytomegalovirus, fungal infections, and noninfectious causes (e.g., pulmonary edema or organizing pneumonia), were considered unlikely based on the clinical course, imaging, and laboratory findings. RT-PCR, reverse transcriptase-polymerase chain reaction

On day 97, peripheral blood stem cell transplantation from a haploidentical sibling donor was performed (designated day 0 post-HSCT). Fludarabine (150 mg/m²) and intravenous busulfan (12.8 mg/kg) were administered as conditioning regimens, and post-transplantation cyclophosphamide, tacrolimus, and mycophenolate mofetil were administered for graft-versus-host disease prophylaxis. At the initiation of the conditioning regimen, cyclosporine was discontinued and a tapering strategy was implemented for corticosteroids. Neutrophil engraftment was observed on day 15 post-HSCT. Hemorrhagic cystitis due to adenoviral infection developed on day 26 post-HSCT, and treatment with ganciclovir was initiated. On day 49 post-HSCT, chest CT was performed because of mild hypoxia, which revealed ground-glass opacities (GGOs) and consolidations in the lower lobes of both lungs. The patient was treated with carbapenem, levofloxacin, vancomycin, and voriconazole. However, respiratory failure progressed rapidly from day 57 post-HSCT, and ventilator support was initiated on day 60 post-HSCT (day 157 after the initial COVID-19 infection). Both SARS-CoV-2 antigen testing and RT-PCR, which were conducted as screening tests during intubation, yielded positive results. The RT-PCR cycle threshold (Ct) value was 18.6, indicating a high viral load suggestive of active infection rather than residual viral RNA from a prior infection. Respiratory specimens collected from the lower respiratory tract after intubation were analyzed using the BioFire FilmArray Respiratory Panel 2.1 (BioFire Diagnostics, Salt Lake City, UT). This detected both SARS-CoV-2 and adenovirus, while other pathogens, such as respiratory syncytial virus (RSV), influenza viruses, Mycoplasma pneumoniae, and Chlamydophila pneumoniae tested negative. Chest CT showed increased pulmonary consolidation, with an air bronchogram presenting a crazy-paving pattern. Laboratory tests, including serum β-D-glucan, Aspergillus galactomannan antigen, and cytomegalovirus pp65 antigen, were all negative, and sputum cultures did not reveal any significant pathogens. The brain natriuretic peptide (BNP) level was only found to be mildly elevated at 150.1 pg/mL, and chest radiography revealed no cardiomegaly when compared with pre-transplant imaging; therefore, pulmonary edema secondary to acute heart failure was considered unlikely. Bronchoscopy was not performed due to the patient's critical respiratory condition. Based on clinical and radiological findings, the patient was diagnosed with COVID-19. The chronological progression of chest CT findings from pre-transplantation to the onset of COVID-19 is shown in Figure 1.

**Figure 2:**
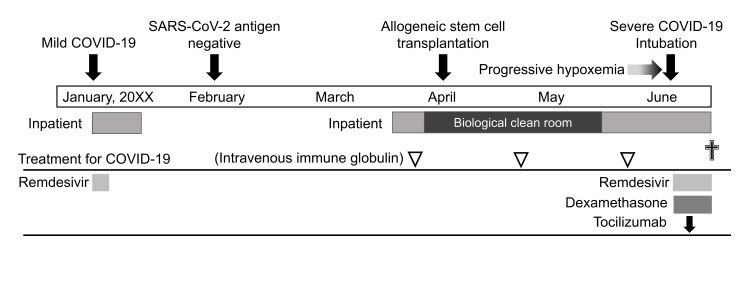
Overall clinical course of this patient. The course is from the onset of the first COVID-19 through allogeneic hematopoietic stem cell transplantation (allo-HSCT) to the development of fetal COVID-19, probably due to the reactivation of SARS-CoV-2. The durations of hospitalization and treatment for COVID-19 are also shown. The patient had severe aplastic anemia before allo-HSCT, accompanied by profound neutropenia and marked lymphopenia. Image credit: Tatsuya Suwabe.

The patient was hospitalized with no visitors and no patients or staff in the same ward who had COVID-19, which indicated possible reactivation of SARS-CoV-2, which had infected him five months earlier. The patient was treated with remdesivir, dexamethasone, and tocilizumab; however, respiratory failure progressed, and the patient eventually died on day 68 post-HSCT. The patient’s clinical course is shown in Figure 2.

## Discussion

Recent studies have reported both viral persistence and reactivation of SARS-CoV-2. Viral persistence is characterized by the prolonged presence of the virus in host cells, where it replicates slowly or at low levels without causing rapid cell death or overt damage [5]. Persistent SARS-CoV-2 infection is thought to result from a combination of impaired immune responses and the formation of tissue reservoirs that support ongoing viral replication, particularly in immunocompromised individuals [5]. Notably, even after viral RNA becomes undetectable by RT-PCR in upper respiratory tract samples, SARS-CoV-2 may persist specifically in the lower respiratory tract [2,6]. In addition to the lower respiratory tract, persistent viral RNA and proteins have also been detected in other anatomical compartments, including the gastrointestinal tract, lymphoid tissues, and central nervous system, thus suggesting the formation of tissue reservoirs that may support continued viral replication in some cases [2,6,7].

In the present case, persistent SARS-CoV-2 infection could not be definitively established. However, the patient did not achieve SARS-CoV-2 antigen negativity until day 27, whereas most individuals tested negative by day 10 after infection [8], suggesting impaired viral clearance. This delayed clearance may be partly attributable to the prolonged corticosteroid therapy for myasthenia gravis, which likely leads to B-cell lymphopenia and hypogammaglobulinemia. While the median duration of RT-PCR positivity is approximately 24 days [9], prolonged infections have been reported in patients with hematologic malignancies receiving B-cell-depleting therapy, with some cases exhibiting RT-PCR positivity lasting over six months [10,11]. Additionally, the patient exhibited sustained lymphopenia before transplantation, indicative of an underlying cellular immunodeficiency. In particular, a reduction in CD4+ T-cell count may have contributed to delayed viral clearance, as previously reported [12].

On the other hand, the viral reactivation is the process by which a latent virus switches to a lytic phase of replication, leading to productive viral replication and the potential spread of the virus [5]. Compared with viral persistence, SARS-CoV-2 reactivation remains poorly understood. Particularly, in the absence of viral genomic data, distinguishing between reactivation and reinfection is highly challenging [2]. Currently, evidence of reactivation is largely limited to a small number of case reports or anecdotal observations [13-16]. Some of these reports involved individuals with profound immunosuppression, similar to the current case [13,14]. Furthermore, a few patients required a higher level of medical care at the time of COVID-19 recurrence than at the time of their initial diagnosis, and these recurrences occurred more than two months after the initial infection [14,15].

Reactivation of various latent viruses is frequently observed after allo-HSCT, primarily due to pre-transplant conditioning regimens and immunosuppressive therapies, compounded by the impaired reconstitution of virus-specific immune responses [17]. In the present case, hemorrhagic cystitis due to adenovirus reactivation occurred before the onset of severe COVID-19. Notably, the clinical course of COVID-19 is more severe at the time of suspected viral reactivation than during the initial episode. Previous studies have implicated macrophages and neutrophils in the pathogenesis of severe SARS-CoV-2 pneumonia and the development of acute respiratory distress syndrome (ARDS) [18,19]. In this patient, a pre-transplant deficiency in these innate immune cells may have attenuated the severity of the initial infection. Conversely, rapid post-transplant expansion of donor-derived neutrophils and macrophages may have contributed to the development of fatal ARDS.

During the allo-HSCT in the present case, routine RT-PCR testing for SARS-CoV-2 was not performed at our institution in asymptomatic patients with negative antigen screening results. In contrast, the updated 2022 guidelines of the American Society for Transplantation and Cellular Therapy (ASTCT) recommend RT-PCR testing for all patients undergoing allo-HSCT, whereas antigen testing is discouraged because of its low sensitivity [1]. In addition to enhanced screening by RT-PCR, potential prophylactic strategies against COVID-19 include vaccination and pre-exposure administration of monoclonal antibodies, such as tixagevimab and cilgavimab. However, their efficacy in patients with persistent SARS-CoV-2 infection remains unclear, as current evidence and guidelines primarily address the prevention rather than the management of established long-term viral persistence [1]. In the present case, intravenous immunoglobulin (IVIG) was administered regularly to treat hypogammaglobulinemia. Previous studies have reported that IVIG may be beneficial in preventing the reactivation of latent viruses, such as cytomegalovirus (CMV) and BK virus, following transplantation [20,21]. However, the efficacy of IVIG in preventing SARS-CoV-2 persistence or reactivation has not yet been established, and further studies are warranted.

After allo-HSCT, COVID-19 was initially difficult to suspect in this case, as the patient remained hospitalized in a protective environment without visitors until the onset of respiratory failure. However, an earlier diagnosis might have been possible through a more careful evaluation of the GGOs observed on chest CT at the onset of mild hypoxia. GGO can be the sole radiographic manifestation in early COVID-19 and may serve as an early indicator of disease progression, particularly in immunocompromised patients [22,23]. The differential diagnosis of GGO includes infectious causes such as Pneumocystis jirovecii pneumonia, viral pneumonia (e.g., RSV, CMV, and SARS-CoV-2), atypical bacterial infections, non-infectious etiologies such as interstitial lung disease, organizing pneumonia, drug-induced lung injury, diffuse alveolar hemorrhage (DAH), pulmonary edema, and malignancies, including lepidic adenocarcinoma and lymphoproliferative disorders [22,23]. In this case, FilmArray Respiratory Panel 2.1, performed after intubation, was instrumental in detecting SARS-CoV-2 and ruling out other respiratory pathogens; however, earlier use might have facilitated timely diagnosis and intervention. Cryptogenic organizing pneumonia (COP), a known late-onset noninfectious pulmonary complication of allo-HSCT, was also considered. However, its typical onset beyond day 100 did not align with the clinical course in this case [24]. As the respiratory failure progressed, chest CT findings evolved from bilateral GGOs to air bronchograms and a crazy-paving pattern, consistent with the well-documented temporal imaging changes observed in COVID-19 pneumonia [23]. Although the crazy-paving pattern is often observed in COVID-19, it is a nonspecific finding that can also be observed in conditions such as DAH and pulmonary alveolar proteinosis [23]. In the present case, CT imaging alone was insufficient to reliably differentiate between these possibilities. Ultimately, the diagnosis of COVID-19 reactivation was supported by a low Ct value on RT-PCR, indicating a high viral load, rapid respiratory deterioration, characteristic imaging findings, and exclusion of alternative etiologies.

At the time of the first infection in this case (December 2022), local surveillance data from the public health center indicated that all of the SARS-CoV-2 cases in our region were of the Omicron variant, with BA.5 accounting for over 95%. A previous study reported a high risk of mortality associated with COVID-19 in patients who underwent allo-HSCT [3]. However, these data were primarily derived from the early pandemic period (early 2020 to early 2021), when the Delta variant predominated. Data on post-Omicron variants, which began to emerge in late 2021, are still limited. Although both the severity and mortality of COVID-19 have decreased in the Omicron era when compared to the pre-Omicron period [25-26], hematologic and immunocompromised patients continue to face a significantly higher risk of severe outcomes than the general population [27-28]. Therefore, COVID-19 remains a critical threat requiring continued vigilance, particularly in patients undergoing allo-HSCT.

This study had several limitations. First, SARS-CoV-2 whole-genome sequencing was not performed, making it impossible to definitively distinguish between viral reactivation and reinfection. Second, RT-PCR testing was not conducted before allo-HSCT, which limited the ability to assess persistent infection. Ideally, lower respiratory tract specimens such as bronchoalveolar lavage fluid should be obtained to better assess potential occult viral reservoirs. Third, the patient’s immune response to SARS-CoV-2 was not evaluated as no antibody testing was performed. Finally, as a single case report, this study could not establish causality or generalize the risk of SARS-CoV-2 reactivation in allo-HSCT recipients. Nevertheless, this case highlights the need for careful pre- and post-transplantation viral monitoring, especially in immunocompromised patients, to ensure safe transplantation during the ongoing COVID-19 era.

## Conclusions

This case highlights the possibility of SARS-CoV-2 reactivation after allo-HSCT, underscoring the ongoing risk of severe or fatal COVID-19 in immunocompromised patients, even in the Omicron era. The findings emphasize the importance of implementing strict RT-PCR-based screening protocols both pre- and post-transplantation, even in asymptomatic patients with negative antigen tests. Furthermore, when respiratory failure occurs early after allo-HSCT in recipients with prior COVID-19, considering reactivation as a differential diagnosis is essential for timely intervention and potentially lifesaving treatment.
